# A Transparent Poly(vinyl alcohol) Ion‐Conducting Organohydrogel for Skin‐Based Strain‐Sensing Applications

**DOI:** 10.1002/adhm.202300076

**Published:** 2023-06-04

**Authors:** Jennie J. Paik, Boonjae Jang, Sunghyun Nam, L. Jay Guo

**Affiliations:** ^1^ Macromolecular Science and Engineering College of Engineering University of Michigan Ann Arbor MI 48109 USA; ^2^ Electrical Engineering and Computer Science College of Engineering University of Michigan Ann Arbor MI 48109 USA

**Keywords:** gel polymer electrolytes, hydrogels, PVA, strain sensors, transparency

## Abstract

The increasing demand for cost‐efficient and user‐friendly wearable electronic devices has led to the development of stretchable electronics that are both cost‐effective and capable of maintaining sustained adhesion and electrical performance under duress. This study reports on a novel physically crosslinked poly(vinyl alcohol) (PVA)‐based hydrogel that serves as a transparent, strain‐sensing skin adhesive for motion monitoring. By incorporating Zn^2+^ into the ice‐templated PVA gel, a densified amorphous structure is observed through optical and scanning electron microscopy, and it is found that the material can stretch up to 800% strain according to tensile tests. Fabrication in a binary glycerol:water solvent results in electrical resistance in the kΩ range, a gauge factor of 0.84, and ionic conductivity on the scale of 10^−4^ S cm^−1^, making it a potentially low‐cost candidate for a stretchable electronic material. This study characterizes the relationship between improved electrical performance and polymer–polymer interactions through spectroscopic techniques, which play a role in the transport of ionic species through the material.

## Graphical abstract

Reported herein is a novel poly(vinyl alcohol) (PVA)‐based ion‐conductive organohydrogel for use as a potentially low‐cost candidate for a stretchable electronic material. This work uses spectroscopic techniques to characterize the relationship between the ionic conductivity and polymer–polymer interactions, which is believed to play a role in the transport of ionic species through the material.

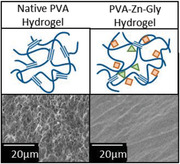

## 1 Introduction

The rising demand for cost‐efficient and user‐friendly wearable electronic devices has led to the emergence of stretchable electronics. However, current wearable devices utilize stiff sensors that perform poorly due to the nonconformal contact with the soft, curvilinear surface of the skin. Next‐generation wearable devices must be conformal and maintain electrical connectivity under duress to avoid negatively impacting sensing performance. While many flexible electronic devices are fabricated on polymeric substrates, such as poly(ethylene terephthalate) (PET), polydimethyl siloxane (PDMS), or polyimides (PI), these substrates are not suitable for stretchable electronics due to their rigidity and low dynamic range.^[^ ^1^, ^2^, ^3^ ^]^ The commercialization of next‐generation wearable devices requires biocompatibility, cost efficiency, and aesthetics in addition to performance. Therefore, a nontoxic, durable, and functional soft material could benefit multiple application sectors and significantly impact society.

Poly(vinyl alcohol) (PVA) hydro‐ and organogel‐based materials are promising materials for biointerfaced applications due to their nontoxicity, cost‐effectiveness, and biocompatibility.^[^ ^4^ ^]^ Flexible hydrogel strain sensors based on PVA have gained significant interest in recent years, with a heavy focus on conductivity, stretchability, and mechanical toughness. Previous strategies for achieving conductivity have involved doping with salts such as sodium chloride,^[^ ^5^, ^6^, ^7^, ^8^, ^9^, ^10^ ^]^ zinc sulfate,^[^ ^11^ ^]^ sodium borate,^[^ ^12^, ^13^, ^14^ ^]^ iron(III) chloride,^[^ ^15^, ^16^ ^]^ calcium chloride,^[^ ^17^, ^18^ ^]^ or lithium chloride.^[^ ^19^ ^]^ Higher gauge factors (GFs) and wider dynamic range can be achieved by using conductive polymers like poly(3,4‐ethylenedioxythiophene) polystyrene sulfonate (PEDOT:PSS)^[^ ^20^ ^]^ or conductive fillers like graphene,^[^ ^9^, ^12^ ^]^ silver nanowires (AgNWs),^[^ ^12^, ^21^ ^]^ or carbon nanotubes (CNTs).^[^ ^12^, ^16^ ^]^ While high performing, these electrically conducting additives can be detrimental to biocompatiblity, flexibility, and aesthetics. PVA networks are formed by repeatedly freezing and thawing an aqueous PVA solution, resulting in amorphous domains interspersed with crystalline domains that act as crosslinking points in the network. These crystalline domains render the gel opaque and impede their ability to dissipate energy under external strain, which is critical to achieve high elongation and high dynamic range.^[^ ^4^ ^]^ For skin‐interfaced applications where transparency, colorlessness, and comfort are important, it is desirable to have a single material possessing multiple properties, rather than a multicomponent or multilayered device. Therefore, we present a transparent, stretchable, ion‐conductive PVA gel. The material's conducting and sensing properties are tuned by the physically bonded polymer network. This work represents a step toward the development of a single, multifunctional material for skin‐interfaced devices.

**Table** **1** summarizes the compositions, mechanical properties, and capabilities of a selection of PVA‐hydrogel‐based strain sensors from recent years, highlighting the versatility of these materials. Despite the diverse strategies employed to develop soft PVA‐based sensors, there is little work on how changes to hydrogen bonding within the PVA matrix impact sensing performance. This study aims to investigate the relationship between polymer–polymer interactions, polymer dynamics, and sensing performance in a completely physically crosslinked PVA–glycerol–zinc salt system. By optimizing glycerol content, we have developed a physically crosslinked PVA‐based hydrogel sensor with good sensitivity (GF 0.89) and high dynamic range (5–300%). High glycerol loading raises the modulus, and reduces conductivity and sensitivity due to increased hydrogen bonding within the gel interfering with ion percolation pathways. Thus, it is necessary to balance mechanical properties and sensing performance in a physically crosslinked gel matrix. Fabrication in a binary glycerol:water solvent renders electrical resistance in the kilo‐Ohm range and ionic conductivity on the scale of 10^−4^ S cm^−1^, making it a potentially low‐cost candidate for a stretchable electronic material.

**Table 1:** Selected recent works on PVA‐based hydrogel sensors

Work	Composition	%T	Ultimate Tensile Strength (UTS) (kPa)	Rupture Strain (%)	E (kPa)	Conductivity (S*cm^‐1^)	GF	Range (%)
This Work	PVA/Glycerol/Zn(NO_3_)_2_	>90%	1000‐3750	200‐800	1.3‐5.75	1.79E‐4‐3.54E‐4	0.54‐1.254	0‐300
Cai et al.^[12]^	PVA/CNT/ Borax/AgNW	No	NA	NA	NA	NA	1.51	2‐100
Liu et al.^[13]^	PVA/Borax/ Dopamine	No	0.45	NA	∼0.4	NA	3.5	500+
Zhou et al.^[5]^	PVA/Hydroxypropyl celulose (HPC)/ NaCl	No	1300	520	590	0.0275	1.31	400+
Jing et al. ^[14]^	PVA/CNF/ CaCl_2_/Borax	>90%	3.5	1900	11.2	0.025	NA	NA
Pan et al.^[6]^	PVA/Glycerol/NaCl	>88%	3100	570.7	550	NA	4.01	0.5‐100
Hu et al.^[11]^	PVA/CNF/ ZnSO_4_	No	790	242	4	0.0032	1.7	200+
Guo et al.^[16]^	PVA/FeCl_3_/CNF/CNT	No	1100	336	119	0.0057	1.39	5‐300
Di et al.^[8]^	PVA/NaCl	No	8030	725	1000	0.0714	0.989	0.2‐400
He et al.^[18]^	PVA/Starch/ Ethylene glycol/Tannic Acid/CaCl_2_	No	1100	608	430	0.013	2.96	3‐150
Bai et al.^[15]^	Styryl‐pyridinium‐PVA/FeCl_3_	Trans‐parent	300	858	NA	0.0029	NA	NA
Wei et al.^[9]^	PVA/ Graphene oxide/ NaCl	No	65	215	0.2	0.0338	2.05	150+
Gong et al.^[20]^	PVA/PEDOT:PSS/Sodium polyacrylate	No	190	289	1970	0.00327	1.1	200+
Liu et al.^[10]^	PVA/CNF/ Zirconium phosphate nanosheets/ glycerol/NaCl	87%	2420	5	541	0.00765	2.46	350+
Zhang et al.^[7]^	PVA/Carboxymethyl chitosan/ Tannic Acid/Glycerol/NaCl/ Al(NO3)_3_	No	2020	570.82	3250	0.0305	2.818	400+
Liu et al.^[17]^	PVA/SA/ Glycerol/ CaCl_2_	96.5%	2290	816	800	0.0208	2.68	500+
Wang et al.^[19]^	PVA/Poly acrylamide/ Polyethylene imine/LiCl	No	500	150	400	NA	1	NA

## 2 Results and Discussion

A binary glycerol/water solvent system has been identified as a potential route to fabricate transparent, ion‐conductive PVA hydrogels. This study investigates the effect of glycerol loading on the tensile, adhesive, conductive, and resistive strain‐sensing properties of a PVA organohydrogel fabricated in a binary glycerol/water system, as shown in **Table** **2**. Our findings have revealed several interesting insights.

**Table 2:** Organohydrogel compositions, sample code, and molar ratios

Sample	Description	*n*OH_PVA_:*n*OH_Gly_:*n*Zn^2+^
PVA	10 wt% PVA in water	1:0:0
GPZ00	10 wt% PVA + Zn^2+^ in water	1:0:0.3
GPZ10	10 wt% PVA + Zn^2+^ in glycerol:water, 10:90	1:1.43:0.3
GPZ15	10 wt% PVA + Zn^2+^ in glycerol:water,15:85	1:2.145:0.3
GPZ20	10 wt% PVA + Zn^2+^ in glycerol:water, 20:80	1:2.85:0.3
GPZ25	10 wt% PVA + Zn^2+^ in glycerol:water, 25:75	1:3.61:0.3
GPZ40	10 wt% PVA + Zn^2+^ in glycerol:water, 40:60	1:5.65:0.3

### 2.1 Effects of Glycerol Loading on Strain Sensitivity and Ionic Conductivity

It is well established in the literature on ion‐conducting gels that ion conductivity correlates with polymer segmental mobility, in which amorphous regions provide ion percolation pathways in addition to diffusion of a liquid‐phase electrolyte through the gel.^[^ ^22^, ^23^ ^]^ One strategy to increase amorphous content in polymer or gel electrolyte systems is to incorporate small, hydrophilic polyols, which increase polymer chain mobility and provide more conductive paths for ions to travel, thereby enhancing ionic conductivity.^[^ ^24^, ^25^ ^]^ Additionally, a high dielectric constant medium is required to break strong Coulombic interactions of oppositely charged ions to produce free ions, and incorporation of a high dielectric constant polyol may be beneficial for sensing applications.^[^ ^26^ ^]^ Due to its relatively high dielectric constant of 40–42, glycerol is effective at reducing the strength of Coulombic attraction among cations and anions of salts and among polymer chains, thereby increasing the liability of the entire system.^[^ ^27^, ^28^ ^]^ A previously published iteration of this material gives a sensor whose electrical resistance operates within the mega‐Ohm regime.^[^ ^21^ ^]^ In this work, the addition of glycerol reduces the resistance to the kilo‐Ohm regime, which is much easier to measure and can facilitate high speed sensing by reducing RC delay.

**Figure 1:**
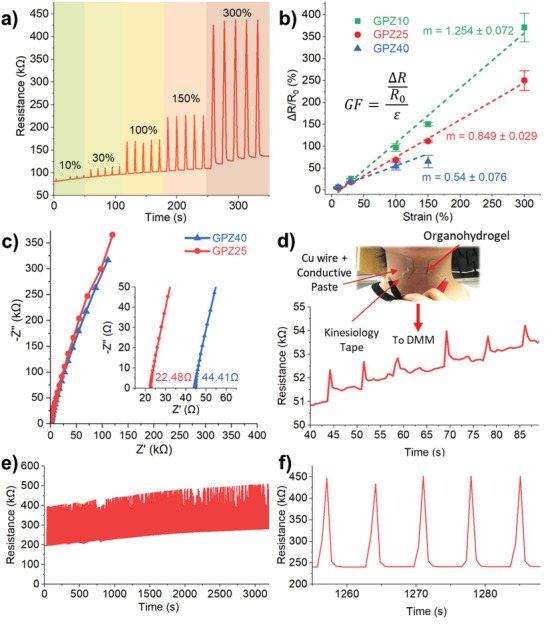
a) Resistance response to different % strain of GPZ25. b) Strain sensitivity of GPZ10, GPZ25, and GPZ40 (*n* = 5). c) EIS of GPZ25 and GPZ40 samples. d) Response of prototype vocal sensor to the word “hello”; inset is schematic of prototype vocal sensor. e) Cyclic fatigue test of 1000 pulls at 50% strain. f) Close‐up of cyclic fatigue response.

**Figure** **1**a demonstrates the increase in resistance response to strains between 10% and 300% for a sample of GPZ25. The GPZ gels demonstrate a mechanoresponsive effect that operates at large strains, which is desirable for sensors requiring not only sensitivity but also dynamic range. This test was repeated for GPZ10 and GPZ40, and the relative % resistance change Δ*R*/*R*_0_ was plotted against the strain % *ε* as shown in Figure 1b. The slope of this plot is the GF, the equation for which shown in the inset of Figure 1b. GPZ gels demonstrate strain sensitivity where increases in resistance are elicited by corresponding strains, from 10% to 300%. As shown in Figure 1b, strain sensitivity decreases with increased glycerol content, indicating that high glycerol loading may increase resistance to ionic migration.^[^ ^17^ ^]^

Electrical impedance spectroscopy (EIS) was used to determine ionic conductivity. Two mechanically robust organohydrogel samples, GPZ25 and GPZ40, were selected and their conductivities were characterized through EIS, as shown in Figure 1c. Sample GPZ25 was found to have a conductivity of 3.54E‐4 S cm^−1^ while sample GPZ40 was found to have a conductivity of 1.79E‐4 S cm^−1^, showing a decrease in ionic conductivity as more glycerol is added and polymer chain mobility is restricted.

To showcase the practical applications of this material, GPZ25 was selected for its optimal balance of sensitivity and elasticity. As shown in Figure 1d, when mounted upon the throat over the hyoid cartilage, a repeated response to the word “hello” was elicited. Additionally, a cyclic fatigue test of 1000 pulls at 50% strain was conducted on GPZ25. Figure 1e shows a relatively consistent response over 1000 pulls. A closeup of this response is shown in Figure 1f.

The conductivity of GPZ is due to ion percolation through amorphous regions in addition to diffusion of the liquid‐phase electrolyte through the network. As both pathways involve ion motion through the network, decrease in polymer mobility can impede ion mobility, thereby affecting response sensitivity. Achieving an ideal gel polymer electrolyte requires balance between desirable mechanical stability and polymer/ion mobility, which can be achieved by tuning the amount of glycerol in this system.

### 2.2 Microstructure Shows Amorphousness after Addition of Zn Salt and Glycerol

**Figure 2:**
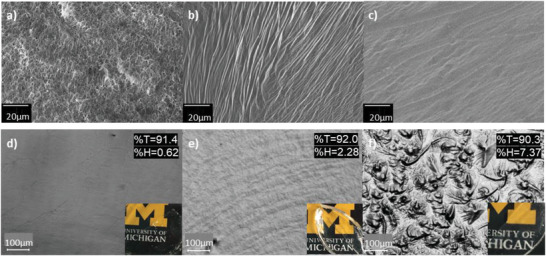
SEM images of a) PVA hydrogel (PVA), b) PVA–Zn hydrogel (GPZ00), c) PVA–Zn‐10% Glycerol hydrogel (GPZ10). Photos showing gel surface optical microscopic images, %transparency, and %haze of d) GPZ10, e) GPZ25, and f) GPZ40 organohydrogels.

PVA networks formed by repeatedly freezing and thawing an aqueous PVA solution are highly crystalline and therefore opaque, limiting their use in applications where transparency is required. Ion‐conducting PVA gels can be made transparent by controlling crystallinity during the freezing–thawing process through fabrication in a binary water/polyol system, which is known to shrink and homogenize the porous PVA structure, thereby increasing transparency.^[^ ^29^, ^30^ ^]^ Pan et al. reported a transparent (>88%) PVA–glycerol–NaCl hydrogel whose conductivity, strong mechanical properties, and hierarchical microstructure arise from the salt‐out properties of NaCl.^[^ ^6^ ^]^ Liu et al. demonstrated that a similar PVA–glycerol–NaCl hydrogel maintained 86% transparency even with the addition of zirconium phosphate nanosheets and cellulose nanofibers (CNFs).^[^ ^10^ ^]^ Another PVA–glycerol–sodium alginate (SA)–CaCl_2_ system developed by X. Liu et al. achieved 96% transparency.^[^ ^17^ ^]^ Though incorporation of polyols is beneficial for transparency, their effects on the sensing performance of ion‐conductive PVA systems have not been sufficiently studied, highlighting the need for further research in this area. In this study, crystallinity reduction of PVA gels was achieved by interrupting PVA–PVA H‐bonding during ice templating through the addition of glycerol and zinc nitrate (Zn(NO_3_)_2_) (Figure S1, Supporting Information). Ice‐templated PVA gels are formed when the frozen sol forms large ice crystals that push the polymer chains together, resulting in the porous structure in **Figure** **2**a.^[^ ^31^, ^32^, ^33^ ^]^ Previous reports of this material have utilized a PVA hydrogel formed in an aqueous Zn(NO_3_)_2_ electrolyte to disrupt crystallinity, finding that an optimal *n*OH:*n*Zn molar ratio of 10:3 of Zn(NO_3_)_2_ caused the porous structure to densify into an irregular, amorphous structure with reduced porosity, as shown in Figure 2b.^[^ ^21^ ^]^

To improve the mechanical, antifreezing, and antidrying properties of GPZ while maintaining the amorphousness, glycerol was incorporated into GPZ.^[^ ^29^, ^30^, ^34^ ^]^ Incorporating glycerol in a weight ratio of 10:90 glycerol:water removes all porosity and shows further densification of this scaly structure, as shown in Figure 2c. Glycerol's strong hydrogen bonding capability allows it to act as an antifreeze by competing with water–water hydrogen bonding during ice formation, thereby preventing the formation of large ice crystals.^[^ ^21^ ^]^ While the formation of this scaly structure is beneficial for stretching enhancement due to the amorphousness, it needs to be balanced with the formation of strong hydrogen bonds, which can increase elastic modulus and reduce rupture strain.^[^ ^34^ ^]^

PVA‐based gel sensors described in previous literature often compromise transparency for performance, which is suboptimal for applications where aesthetics are of concern. However, by disrupting the formation of light‐scattering crystallites with glycerol and Zn(NO_3_)_2_, the gel can be made transparent (%*T* > 90%; Figure S2, Supporting Information) while retaining sensing functionality. Figure 2d–f demonstrates the glycerol loading effect on transparency and wrinkling. Increasing glycerol content results in increased wrinkling, increased gel shrinkage during fabrication, and increased haze due to the light‐scattering wrinkles on the surface of the gel.

### 2.3 Mechanical and Adhesive Properties of Organohydrogel Correlate with Glycerol Loading Levels

Current strategies for improving electrical performance in gel sensors involve the use of conductive polymers or fillers, which impart a strong color to the device, limiting their use in applications that require transparency. To increase sensor response without the use of conducting materials, the polymer matrix itself must be soft enough to aid in ion conduction. Flexibility arises from polymer segmental mobility and is critical for increasing sensor response without the use of conducting materials. Flexible moieties or dynamic bonds are commonly employed for softness, while hierarchical morphologies, crosslinked double networks, or structural nanomaterials such as CNFs are often used to maintain mechanical strength.^[^ ^10^, ^11^, ^14^, ^16^ ^]^ X. Liu et al.’s PVA–glycerol–SA–CaCl_2_ network consists of dynamic hydrogen and ion–ligand bonds capable of dissipating energy.^[^ ^17^ ^]^ Y. Liu et al. reported a soft–hard network gel that uses PVA and poly(vinyl pyrrolidone) as the soft, flexible portions while cellulose nanocrystal (CNC)–Fe^3+^ complexes serve as the hard, tough crosslinkers.^[^ ^35^ ^]^ S. Liu et al. achieved a GF of 3.5 in an ultrasoft gel, which is outstanding among ion‐conductive PVA hydrogels without the use of conducting nanomaterials.^[^ ^13^ ^]^ Non‐covalent interactions such as hydrogen bonds, which can rupture and re‐form as the material is strained, are crucial to construct stretchable gels with energy dissipation ability, dynamic regulation, and fast response, but these systems are not yet sufficiently studied.^[^ ^17^ ^]^

Tensile properties of the GPZ gels were measured to determine whether the addition of glycerol affected the bulk properties through microstructure or chemical interaction changes. Native PVA hydrogels are physically crosslinked by the formation of folded polymer crystallites formed through the freezing–thawing process.^[^ ^33^ ^]^ These crystalline domains are interspersed by amorphous domains that are responsible for the flexible and extensible qualities of PVA hydrogels.

**Figure 3:**
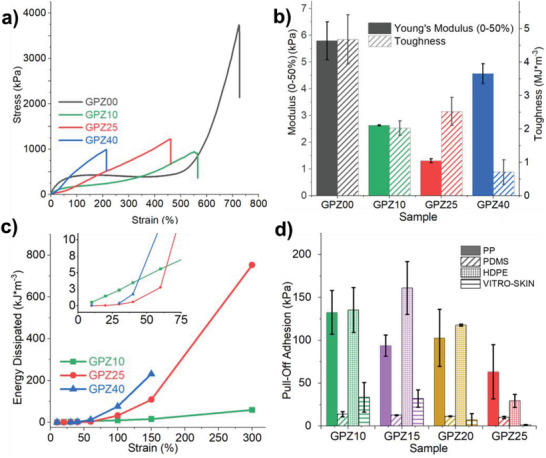
a) Stress–strain curves of PVA–Zn–glycerol gels at varying glycerol:water ratios (*n* = 3). b) Young's modulus (0–50%, kPa; *p* = 2.9 × 10^−5^) and toughness (MJ m^−3^; *p* = 2.5 × 10^−4^) of GPZ gels (*n* = 3). c) Energy dissipating properties of GPZ organohydrogels from 10% to 300% strain; the inset shows energy dissipation at a strain of <60%. d) Pull‐off adhesion (kPa) of PVA organohydrogel to LSE substrates (PP, *p* = 0.29; PDMS, *p* = 0.26; HDPE, *p* = 0.01; VITRO‐SKIN, *p* = 0.0065) at different glycerol:water ratios (*n* = 3).

The inclusion of Zn(NO_3_)_2_ disrupts the formation of these crystallites and results in a transparent, amorphous gel with reduced crystallinity.^[^ ^21^ ^]^ As shown in **Figure** **3**a, under tensile strain, GPZ00 has a short elastic response regime, long plastic deformation regime, and undergoes strain hardening before fracture. The plastic deformation occurs as the loosely associated PVA chains are allowed to slide against each other, and irreversible strain hardening occurs as the stretched chains realign and recrystallize along the tensile axis. As glycerol content is increased, the organohydrogel behaves more like an elastic material, developing a linear stress–strain response. The reduced elongation and elastic behavior are likely due to increased hydrogen bonding within the material by glycerol, which prevents the chain slippage that occurs in the plastic deformation regime of GPZ00.^[^ ^17^ ^]^

The Young's modulus of the hydrogels was determined through linear fitting of the stress–strain curve from 0 to 50% strain. Figure 3b shows the decrease in Young's modulus from 0 to 50% strain as glycerol content is increased from 0:100 to 10:90 glycerol:water, after which the plasticization effect by the glycerol is overtaken by the high amount of hydrogen bonding between PVA chains and glycerol.

The baseline increase across repeated 300% pulls shown in Figure 1a is likely due to the hysteresis at high strain caused by energy dissipation through hydrogen bond breaking; however, strains up to 150% do not exhibit this behavior. To investigate the energy dissipation abilities of this material, hysteresis tests were performed by straining each piece multiple times (Figure S3, Supporting Information). Most mechanosensing applications take place at relatively small strains; however, to investigate the full range, strains up to 300% were investigated. Figure 3c shows that for GPZ25 and GPZ40 a significant amount of hysteresis is observed at strains greater than 60%. At these strains, the amount of energy dissipated with each subsequent strain cycle increases with the amount of glycerol added due to increased abundance of hydrogen bonds to rupture.

The effect of glycerol on hysteresis is more pronounced below 60% strain, as shown in the inset of Figure 3c. Increased synergistic hydrogen bonding within the material delays the onset of hysteresis for GPZ25 and GPZ40, indicating that the plasticization effect is canceled out by the increased hydrogen bonding at these loading levels. Conversely, GPZ10 experiences significant hysteresis at very small strains, as this amount of glycerol does not provide sufficient hydrogen bonding to overcome the interrupted PVA–PVA interactions.^[^ ^17^ ^]^

For this system, strain sensitivity and recoverability are in opposition. To have a wide dynamic range, fast recovery after deformation is required; therefore, the material should resist hysteresis, but it is unavoidable for noncovalently crosslinked networks with large dynamic range. Conversely, a lower Young's modulus is beneficial for strain sensitivity. GPZ10 has the highest sensitivity of the tested samples, but the lowest strain recovery ability (Figure S4, Supporting Information). Thus, GPZ25 is a more suitable strain sensor balancing sensitivity and recovery. For GPZ25, the usable elastic deformation region appears to be from 0 to 150% strain.

Preliminary results show adhesion to skin for low glycerol‐content organohydrogels due to their softness. In addition, hydrogen bonding between PVA and skin surface amines can improve adhesion. Pull‐off adhesion to low surface energy (LSE) substrates was studied to predict glycerol loading effects on skin adhesion. As GPZ40 is too stiff to form good contact with substrates, glycerol loadings between 10 and 25 wt% were investigated. Increasing glycerol content from 10% to 25% shows a reduction in adhesion to high‐density polyethylene (HDPE) and polypropylene (PP), likely due to a reduction in the amount of surface PVA hydroxyls and reduced wetting‐out capability of the higher‐modulus gel. To observe the effect of substrate surface energy on organohydrogel adhesion, the pull‐off adhesion of the GPZ organohydrogel to several low surface energy substrates was studied including HDPE, PP, silicone, and VITRO‐SKIN with N19 topography, shown in Figure 3d. Optimal adhesive strength appears to be between 10 and 15 wt% glycerol. This optimal adhesion strength is likely related to the ability of the gel to form good contact with the surface, since the low glycerol gels are softer. The elastic response required for recoverability opposes the softness required for adhesion as well. These opposing qualities are less than ideal, which leaves room for future work to achieve both.

### 2.4 Mechanical Property Changes Are Due to Hydrogen Bonding within Organohydrogel

**Figure 4:**
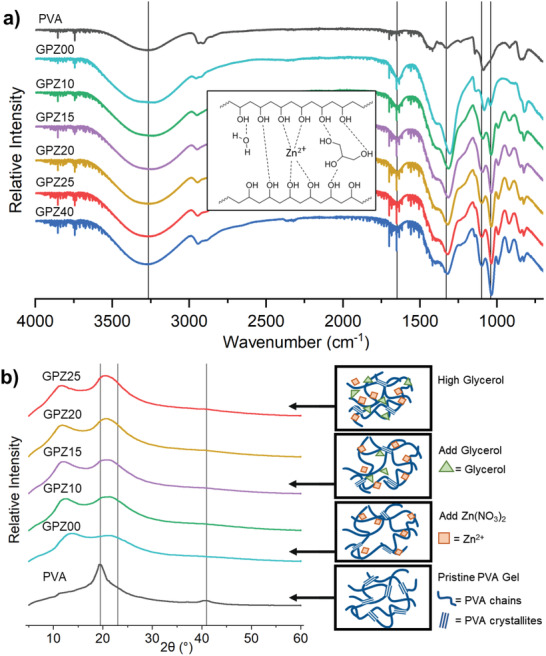
a) FTIR spectra of samples with offset. Inset shows possible intermolecular interactions within organohydrogel. b) XRD of PVA and GPZ samples. Inset shows proposed model of how glycerol loading affects the macromolecular behavior in PVA–Zn–glycerol hydrogels.

To investigate the effect of glycerol loading on hydrogen bonding interactions, Fourier transform infrared (FTIR) spectroscopy was performed on lyophilized samples. As shown in **Figure** **4**a, several major bands corresponding to changes in the hydrogen bonding were identified (Figure S5, Supporting Information). In the FTIR spectrum of PVA, the band at 3271 cm^−1^ corresponds to an intermolecular O—H stretch. Low glycerol loading (GPZ10) lowers the O—H stretch peak to 3245 cm^−1^, indicating the formation of H‐bonds between glycerol and PVA. Table 1 lists the molar ratios of PVA hydroxyls to glycerol hydroxyls (*n*OH_PVA_:*n*OH_Glycerol_). GPZ10 has a *n*OH_PVA_:*n*OH_Glycerol_ = 1:1.43, indicating that the moles of PVA OH are about equal to the moles of glycerol OH. As more glycerol is introduced and *n*OH_PVA_:*n*OH_Glycerol_ increases to 1:5.65, the band shifts to a higher wavenumber closer to that of glycerol, indicating that more PVA–PVA bonds are broken as PVA–glycerol interactions are formed, shifting the peak toward the O—H stretch value for pure glycerol, which is higher than that of PVA.^[^ ^36^, ^37^, ^38^, ^39^, ^40^ ^]^ Similar behavior is observed in for the band at ≈1100 cm^−1^ corresponding to the C—C—O stretch of a secondary alcohol, which blueshifts with increased glycerol loading as PVA–PVA hydrogen bonds are switched to PVA–glycerol hydrogen bonds.^[^ ^36^, ^37^, ^38^, ^39^, ^40^ ^]^

To better observe the changes in hydrogen bonding due to glycerol, we studied the fingerprint band at ≈1040 cm^−1^ corresponding to the C—C—O stretch of a primary alcohol. In pristine PVA, there are very few primary alcohols (occupying hydrolyzed chain ends) compared to secondary alcohols, whereas the majority of alcohol groups in glycerol are primary alcohols. Changes to the primary alcohol C—C—O peak allow us to monitor hydrogen bonding within the organohydrogel as glycerol loading is increased. The primary alcohol C—C—O stretch is a shoulder peak in the IR spectrum of PVA and full peak in the IR spectrum of GPZ00 at 1042 cm^−1^. This is likely due to Zn(NO_3_)_2_ interrupting PVA–PVA interactions, liberating chain end defects that plasticize the gel as shown in the stress–strain curve. After adding glycerol, this peak redshifts to 1038 cm^−1^, indicating that the primary alcohols in glycerol are interacting the with alcohol groups of PVA. Increased glycerol causes the primary alcohol C—C—O band to become more pronounced, owing to the increased presence of primary alcohol groups. The peak shifts further to 1036 cm^−1^, indicating that these primary alcohol groups are participating in hydrogen bonding.

The X‐ray diffraction (XRD) spectrum for PVA is well known and contains three distinct peaks: a crystalline peak around 2*θ* = 18°–21° corresponding to the [10$\bar{1}$] lattice direction, an amorphous peak around 2*θ* = 23°, and a compound peak around 2*θ* = 41°.^[^ ^41^, ^42^, ^43^ ^]^ In addition, residual water has a peak around 2*θ* = 30°. As shown in Figure 4b, the addition of Zn(NO_3_)_2_ and glycerol into the hydrogel drastically broadens the crystalline peak, which denotes a reduction in crystallite size. **Table** **3** shows the apparent crystallite dimensions of each sample; PVA crystallites are roughly 5.93 nm which is on the order of other PVA crystallite sizes reported in the literature.^[^ ^41^ ^]^ The crystallite dimensions decrease with the addition of Zn(NO_3_)_2_ and glycerol, but begin to increase as more glycerol is added. As more glycerol is added, the intensity of the crystalline peak increases. At high loading levels of glycerol, increase in the peak at 2*θ* = 20° is seen, likely due to stronger PVA–glycerol interactions. This correlates to the decrease in elongation and increase in modulus as shown by the mechanical tests.

**Table 3:** Crystalline properties of PVA and GPZ organohydrogels calculated from XRD

Sample	2 ****θ**** [°]	*d*‐spacing [nm]	Apparent crystallite dimensions [nm]
PVA	19.459	0.46	5.93
GPZ00	17.03	0.52	2.57
GPZ10	20.55	0.43	1.36
GPZ15	20.64	0.42	1.57
GPZ20	21.08	0.43	2.28
GPZ25	20.73	0.43	2.29

Based on the tensile, XRD, and FTIR data, we propose a model of how glycerol interacts with the other components in the hydrogel and affects ion mobility, as shown in the inset of Figure 4b. As the transport of ions through GPZ requires high polymer segmental mobility, immobilizing the polymer through a high crosslink density or strong intermolecular interactions will reduce ion conductivity by increasing resistance to ionic migration.^[^ ^17^ ^]^ Native PVA hydrogels are physically crosslinked through the formation of crystalline domains that can be observed using XRD. Disruption of PVA–PVA interactions upon addition of ions prevents the formation of significantly large crystallites, giving an amorphous structure that aids in ionic conduction. Glycerol promotes intermolecular interactions and acts as a physical crosslinker for PVA without significantly increasing crystallinity; however, at high glycerol loadings, PVA–glycerol and glycerol–glycerol interactions dominate, inducing shrinkage, wrinkling, stiffness, and inhomogeneity that eventually degrade the material's mechanical properties and ionic conduction as well. At glycerol loadings where moles of glycerol —OH far exceed moles of PVA —OH, only one or two —OH on each glycerol molecule can participate in H‐bonding with PVA; thus, a critical point of crosslinking is reached and there may be some free glycerol molecules present, which introduce inhomogeneity and may degrade sensor properties. Through careful modulation of glycerol content, the mechanical properties of this organohydrogel can be tuned while maintaining amorphousness and transparency, which is crucial for the engineering of strain sensors.

## 3 Conclusion

Flexible electronics is positioned to be a huge market in the Internet of Things era, where electronic devices can be integrated easily into robotics, clothing, furniture, and even the human body. To gain a deeper understanding of these materials, it is crucial to investigate the relationship between polymer interactions and electrical performance. This study examines the development of a transparent, strain‐sensitive organohydrogel, and its correlation between glycerol loading and electrical performance were investigated. Intermolecular interactions were studied through FTIR and XRD, revealing that as glycerol content is increased, hydrogen bonding within the material increases, resulting in enhanced bulk material elasticity and strain recovery while maintaining amorphousness. However, the increased hydrogen bonding adversely effects strain sensitivity and ionic conductivity, as studied through resistance changes and electrical characterization. A prototype device capable of detecting strain changes induced by movement of the hyoid cartilage during speech was fabricated from a material that balanced these principles. This material has electrical resistance in the kilo‐Ohm range and ionic conductivity on the scale of 10^−4^ S cm^−1^, making it a potentially low‐cost candidate for a stretchable electronic material.

## 4 Experimental Section

### Materials

Poly(vinyl alcohol) (molecular weight (*M*_W_) 89–98 kDa, >99% hydrolysis), glycerol (*m*_w_ = 92.094 g mol^−1^), and zinc nitrate hexahydrate (Zn(NO_3_)_2_·6H_2_O, *m*_w_ = 297.5 g mol^−1^) were purchased from Sigma–Aldrich (St. Louis, MO). Water‐based electric paint (55 Ω sq^−1^ at 50 µm film thickness) was purchased from Bare Conductive (London, UK). HDPE, PP, and silicone sheeting were purchased from McMaster‐Carr (Elmhurst, IL). VITRO‐SKIN with N19 topography was purchased from Florida Suncare Testing Inc., IMS Division (Bunnell, FL). Milli‐Q water (18 MΩ) was used throughout this experiment. All chemicals were used without further purification.

### Hydrogel Fabrication

The gel precursor solution was prepared by dissolving 1 g of PVA in glycerol/water solutions of ratios 0/100, 10/90, 15/85, 20/80, 25/75, or 40/60 wt% to create 10 g of a 10 wt% PVA solution in glycerol/water. Solutions were stirred vigorously for 1 h at 80 °C, then left to cool to room temperature. Upon cooling, 2.03 g of Zn(NO_3_)_2_·6H_2_O was added and stirred at room temperature for 1 h. The solutions were then sonicated for 5 min and left closed overnight to degas. Table 1 shows the molar ratios of PVA hydroxyls to glycerol hydroxyls to Zn^2+^, showing that the addition of glycerol affects hydrogen bonding between polymer chains.

Organohydrogels were prepared by pouring 6 g of solution into a circular polystyrene mold measuring 60 mm in diameter. Gels were then frozen for 24 h at −20 °C and left to thaw for 1 h at 20 °C. This cycle was repeated two more times for the glycerol‐free samples; then all gels were conditioned at 20 °C and 20% relative humidity (RH) for 18 h. A schematic of this entire process is shown in Figure S1 (Supporting Information). Gels were stored in closed Petri dishes sealed with parafilm.

### Optical and Scanning Electron Microscopy

Top‐down optical microscope photos were taken using a Nikon Optiphot. Inverted microscope photos were taken using a Nikon Eclipse TE300 inverted fluorescence microscope. Photos were acquired and measurements were made using the AmScope software.

Scanning electron microscopy (SEM) was performed using a JEOL JSM‐6390. Samples were lyophilized using a Labonco vacuum freeze–drying apparatus. Samples were frozen in liquid nitrogen for 5–10 min immediately prior to lyophilization and lyophilized for 3 days to remove as much water as possible. Samples were then coated with Pt metal for discharging using a Denton vacuum sputter coater for 180 s. SEM Beam voltage was kept low, at 5 kV, to minimize damaging the sample.

### Mechanical and Adhesive Characterization

All mechanical pull‐off adhesion and testing characterizations were performed on a KJ‐1065B Universal testing machine (Dongguan Keijan Instrument Co., LTD). Measurements were performed in triplicate then averaged. Pull‐off adhesion was performed on 10 mm × 10 mm samples on various substrates, including glass, HDPE, PP, PDMS, and VITRO‐SKIN with N19 topography. Tests were performed at ambient conditions unless otherwise noted. Crosshead speed was 50 mm min^−1^.

### FTIR and XRD

XRD was performed on lyophilized hydrogel samples using Bragg–Brentano analysis in a transmission style sample holder. Apparent crystallite dimensions were calculated using the Scherrer formula

(1) $$D_{\mathrm{hkl}}=\frac{k*\lambda }{\beta *\cos\theta }$$

where *D_hkl_* is the apparent crystalline direction along a given lattice direction, *k* is a constant (0.89 rad), *λ* is the X‐ray wavelength (0.154 nm from Cu K*α*), *β* is the full width at half maximum (FWHM) of the crystalline peak, and *θ* is the Bragg angle. Peak deconvolution and integration were performed using OriginPro 2022.

FTIR was performed on lyophilized hydrogel samples using a Nicolet iS20 Fourier transform infrared Spectrometer. Baseline corrections and peak picking were performed using OriginPro 2022 and Python.

### Electrical Characterization

Coin cells were prepared for two‐point EIS in an argon glove box. Briefly, a 20 mm diameter sample of 1 mm thick hydrogel was punched using a die and sandwiched between the steel cathode pan, a steel disk, a steel spacer spring, and the steel anode pan. The entire assembly was crimped together forming the coin cell. The coin cell was placed in a sample holder clamp to ensure good contact and EIS was performed using a Solartron electrical impedance spectrometer from 0.1 to 5000 Hz at an amplitude of 10 mV. Using the relationship

(2) $$\frac{l}{S}=\;k$$

where *l* is the length between plates (hydrogel thickness) and *S* is the cross‐sectional area of cell, the coin cell constant *k* was calculated to be 0.00796 cm^−1^.^[^ ^44^ ^]^ From there, the relationship

(3) $$R_{s}=\;\rho *k$$

was used to calculate resistivity *ρ* in Ω cm, where *R*_s_ is the solution resistance (*x*‐intercept of −*Z*″ vs *Z*′ plot, shown in the inset of Figure 1c).^[^ ^44^, ^45^ ^]^ The resistivity value was then used to calculate ionic conductivity *C* through the relationship

(4) $$C\;=\frac{1}{\rho }\;$$

Strain sensing devices were assembled using a 10 mm × 25 mm × (≈0.5) mm piece of hydrogel. Copper wires were connected 5 mm from each end using Bare Conductive Electrical Paint. Strain testing was performed on a linear actuator with a stepper motor running at 21 Hz programmed by PalPC software. Hydrogel was fixed onto the linear actuator 5 mm from each end, and copper wires were connected using alligator clips to a Keithley 2420 Digital Multimeter. NI Labview was used to collect resistance data. Cyclic tests were performed in the same manner. Experiments involving human subjects were performed with the full, informed consent of the volunteers, who are also the authors of this manuscript. Vocal sensor testing was performed using the same circuitry, with the hydrogel being affixed onto a volunteer's throat where the thyroid cartilage protrudes using kinesiology tape.

### Haze Measurements

Haze measurements were performed on a Perkin‐Elmer Lambda 850 UV–vis spectrophotometer outfitted with a 150 mm InGaAs integrating sphere module. Transmittance spectra for total reflectance and scattering were collected for samples of 1 mm thick. The area under the curves in the range of visible light (380–700 nm) was used to calculate total transmittance *T*_t_ and diffuse transmittance *T*_d_

(5) $$T_{t}=\frac{T_{2}}{T_{1}}\;$$

(6) $$T_{d}=\frac{T_{4}-T_{3}\left(\frac{T_{2}}{T_{1}}\right)}{T_{1}}\;$$

where *T*_1_ and *T*_2_ are the areas under the total reflectance curves for the instrument and the sample respectively, while *T*_3_ and *T*_4_ are the areas under the scattering curves for the instrument and sample, respectively.

### Statistical Analysis

Data with error bars were represented as mean ± standard deviation calculated on a minimum of three independent samples using Microsoft 365 Excel software. Error bars represented standard deviation. A one‐way analysis of variance (ANOVA) test was performed on Microsoft 365 Excel where applicable. Differences were considered significant at *p* < 0.05. Curve fitting, FTIR baselining, XRD peak deconvolution, and curve integration operations were performed in OriginPro 2022.

## Conflict of Interest

The manuscript content is part of a US provisional patent application recently filed by the University of Michigan.

## Supporting information

Supporting Information

## Data Availability

The data that support the findings of this study are available from the corresponding author upon reasonable request.
