# Supplementary material for: A Transparent Poly(vinyl alcohol) Ion‐Conducting Organohydrogel for Skin‐Based Strain‐Sensing Applications
Source: Adv Healthc Mater. 2023 Jun 4;12(22):2300076. doi: 10.1002/adhm.202300076 (PMC11469185; doi:10.1002/adhm.202300076)
Supplement: Supplementary file 1 — Supporting Information [file ADHM-12-2300076-s001.pdf]

ADVANCED
HEALTHCARE
MATERIALS

Supporting Information

for *Adv. Healthcare Mater.*, DOI 10.1002/adhm.202300076

A Transparent Poly(vinyl alcohol) Ion-Conducting Organohydrogel for Skin-Based
Strain-Sensing
Applications

*Jennie J. Paik, Boonjae Jang, Sunghyun Nam and L. Jay Guo**

Supporting Information For:

A Transparent Poly(Vinyl Alcohol) Ion-Conducting Organohydrogel for Skin-Based Strain-Sensing Applications

*Jennie J. Paik, Boonjae Jang, Sunghyun Nam, L. Jay Guo**

J. J. Paik, B. Jang, S. Nam, L. J. Guo

Macromolecular Science and Engineering, College of Engineering, University of Michigan

Ann Arbor, Michigan 48109, United States.

L. J. Guo

Electrical Engineering and Computer Science, College of Engineering, University of Michigan

Ann Arbor, Michigan 48109, United States.
E-mail: guo@umich.edu

Table of Contents

I. Fabrication schematic of ice templating method for making PVA organohydrogels…....S1
II. Transmittance and scattering spectra of GPZ organohydrogels……………....…..….....S2
III. Hysteresis curves of GPZ organohydrogels..S3
IV. Strain response at 30% of GPZ organohydrogel strain sensors...S4
V. FTIR Spectra close-ups of peaks of interest..S5

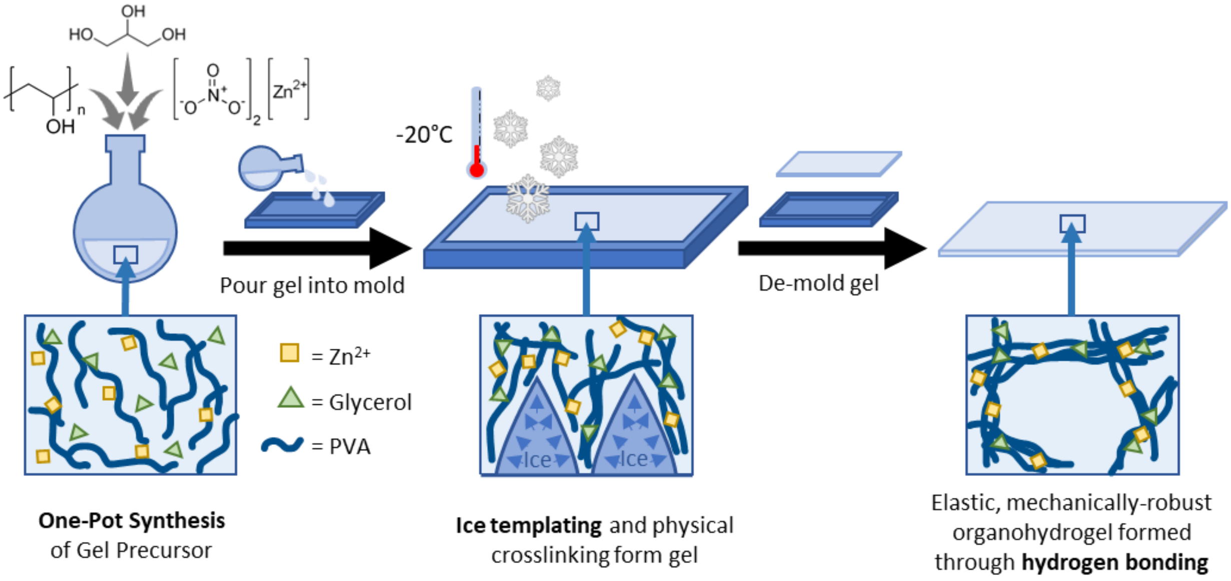

Figure S1. Fabrication schematic of ice templating method for making PVA organohydrogels.

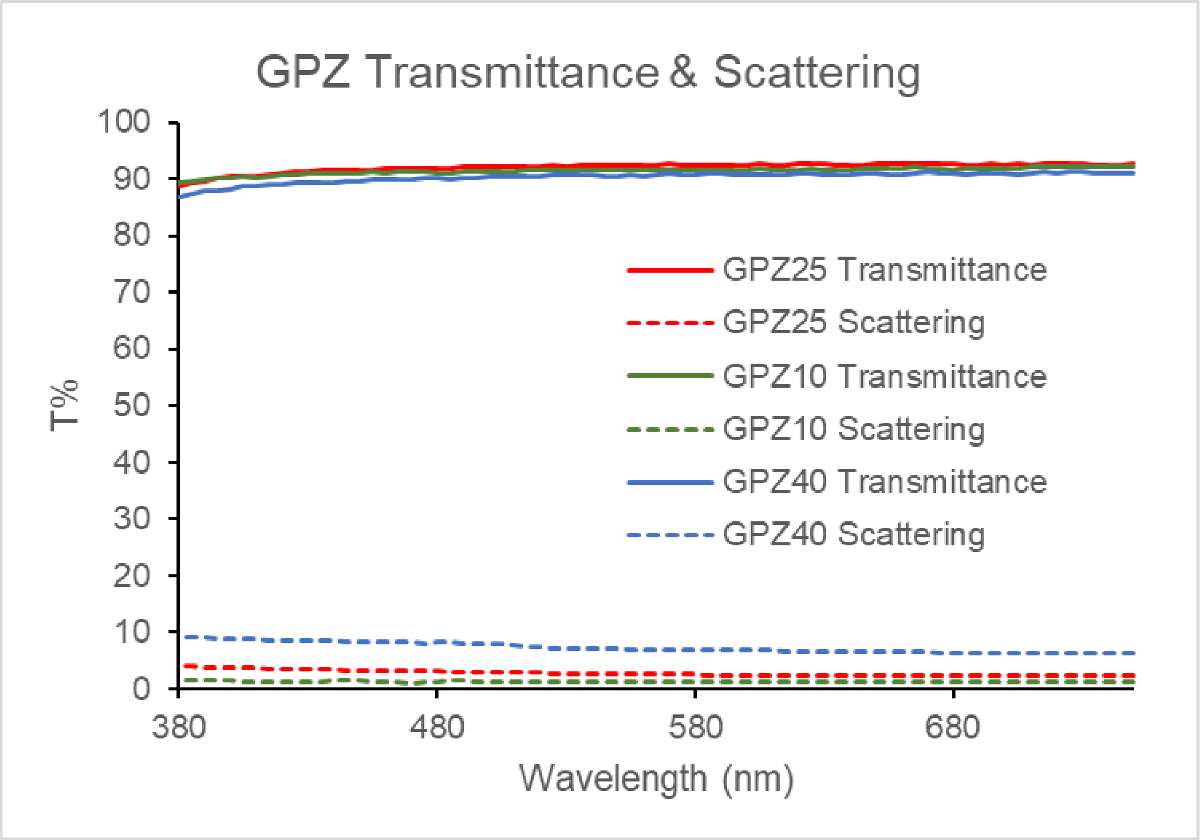

Figure S2. Transmittance and Scattering spectra of GPZ organohydrogels. Used in calculating haze.

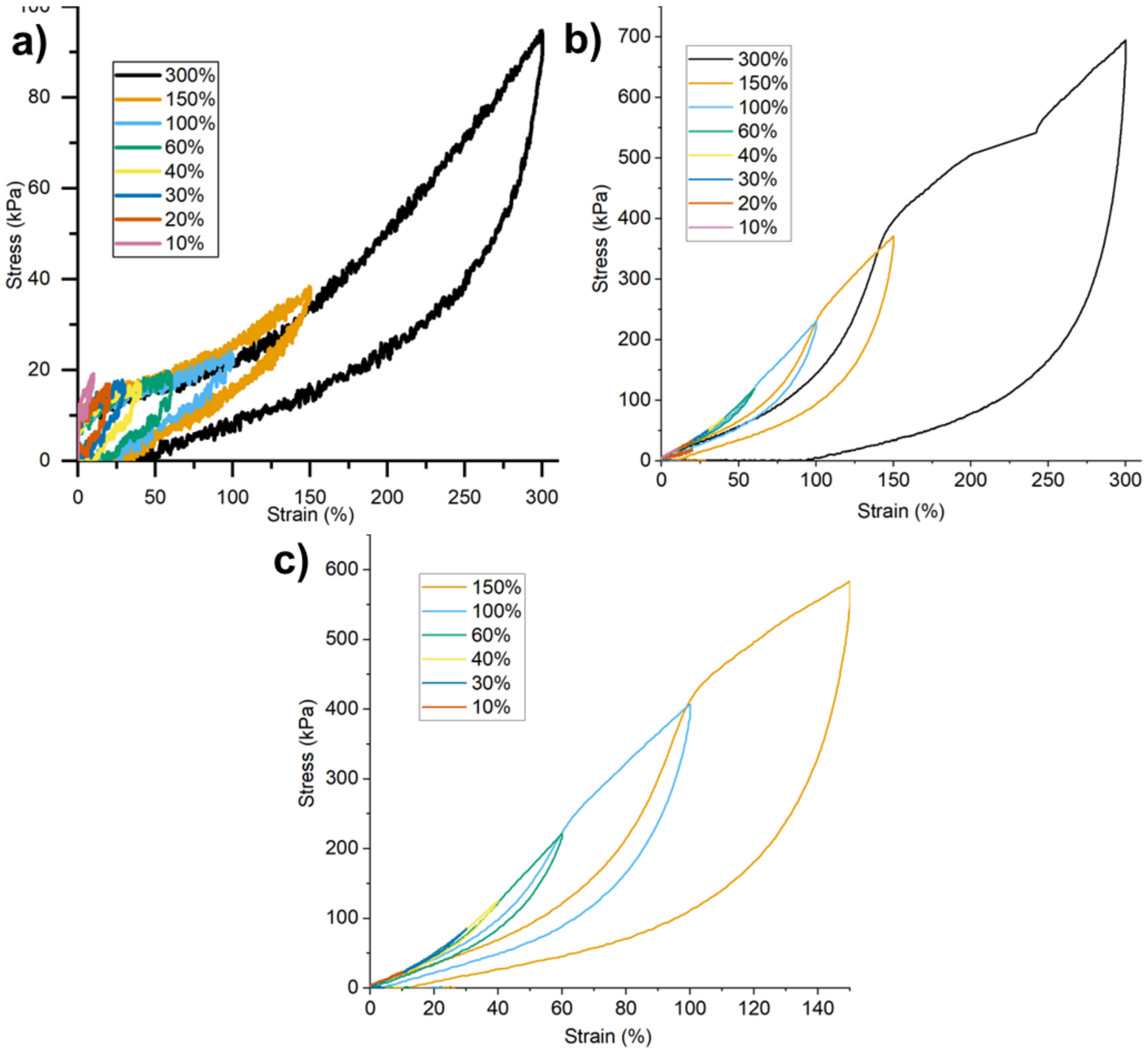

Figure S3. Hysteresis curves of a) GPZ10 b) GPZ25 and c) GPZ40 between strains of 10-300%.

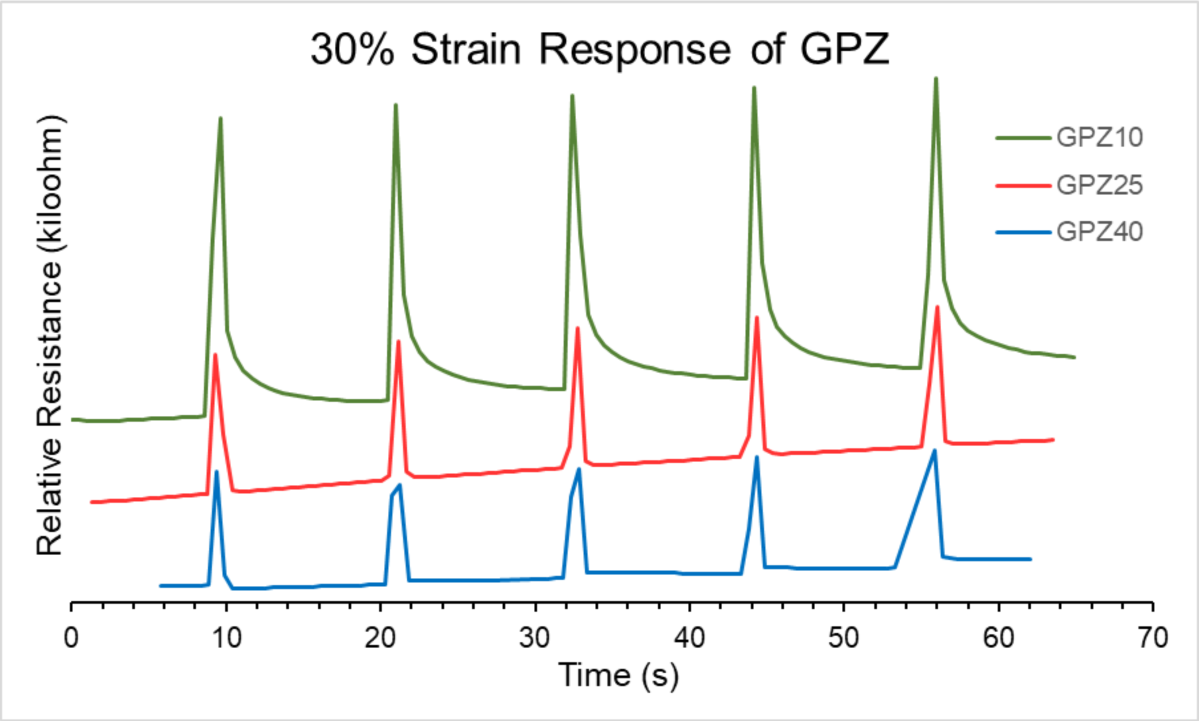

Figure S4. Strain response at 30% of GPZ organohydrogels. Higher glycerol loading, which is associated with an elastic response, demonstrates faster recovery after strain.

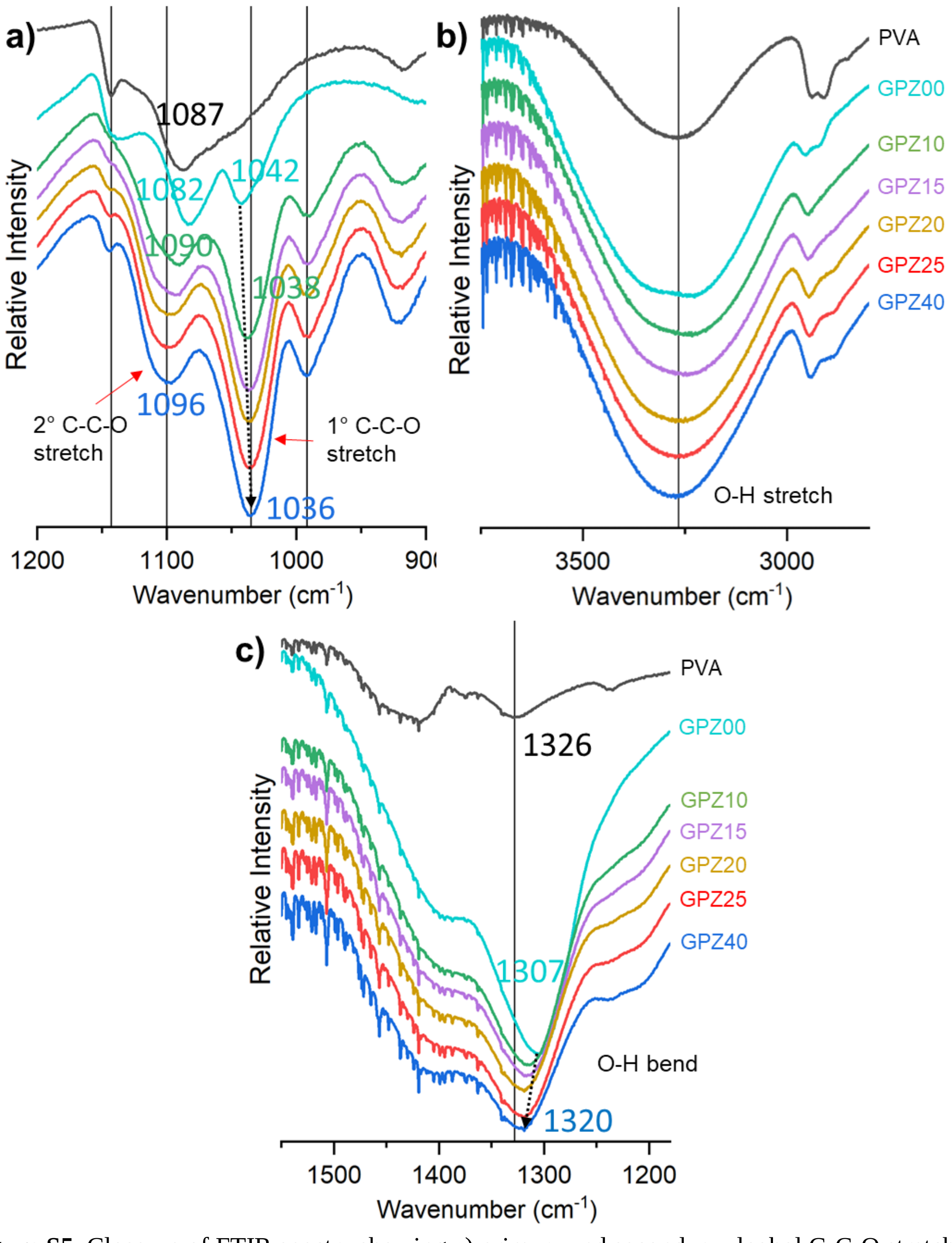

Figure S5. Close-up of FTIR spectra showing a) primary and secondary alcohol C-C-O stretches b) O-H stretch c) O-H bend
